# Evaluation of the Cerebrospinal Fluid Flow Dynamics with Microvascular Imaging Ultrasound in Infants

**DOI:** 10.3390/children10020245

**Published:** 2023-01-30

**Authors:** Luis Octavio Tierradentro-Garcia, Levy Onyango, Rebecca Dennis, Colbey W. Freeman, Sophie Haddad, Brandi Kozak, Misun Hwang

**Affiliations:** 1 Department of Radiology, Children’s Hospital of Philadelphia, Philadelphia, PA 19104, USA; 2 Department of Radiology, Perelman School of Medicine, University of Pennsylvania, Philadelphia, PA 19104, USA

**Keywords:** microvascular imaging, ultrasound, cerebrospinal fluid, hydrocephalus, intraventricular hemorrhage

## Abstract

Purpose: Microvascular imaging ultrasound (MVI) can detect slow blood flow in small-caliber cerebral vessels. This technology may help assess flow in other intracranial structures, such as the ventricular system. In this study, we describe the use of MVI for characterizing intraventricular cerebrospinal fluid (CSF) flow dynamics in infants. Materials and methods: We included infants with brain ultrasound that had MVI B-Flow cine clips in the sagittal plane. Two blinded reviewers examined the images, dictated a diagnostic impression, and identified the third ventricle, cerebral aqueduct, fourth ventricle, and CSF flow direction. A third reviewer evaluated the discrepancies. We evaluated the association of visualization of CSF flow as detectable with MVI, with the diagnostic impressions. We also assessed the inter-rater reliability (IRR) for detecting CSF flow. Results: We evaluated 101 infants, mean age 40 ± 53 days. Based on brain MVI B-Flow, a total of 49 patients had normal brain US scans, 40 had hydrocephalus, 26 had intraventricular hemorrhage (IVH), and 14 had hydrocephalus+IVH. Using spatially moving MVI signal in the third ventricle, cerebral aqueduct, and fourth ventricle as the criteria for CSF flow, CSF flow was identified in 10.9% (n = 11), 15.8% (n = 16), and 16.8% (n = 17) of cases, respectively. Flow direction was detected in 19.8% (n = 20) of cases; 70% (n = 14) was caudocranial, 15% (n = 3) was craniocaudal, and 15% (n = 3) bidirectional, with IRR = 0.662, *p* < 0.001. Visualization of CSF flow was significantly associated with the presence of IVH alone (OR 9.7 [3.3–29.0], *p* < 0.001) and IVH+hydrocephalus (OR 12.4 [3.5–440], *p* < 0.001), but not with hydrocephalus alone (*p* = 0.116). Conclusion: This study demonstrates that MVI can detect CSF flow dynamics in infants with a history of post-hemorrhagic hydrocephalus with a high IRR.

## 1. Introduction

Microvascular imaging ultrasound (MVI) is an advanced Doppler technology that allows the detection of slow blood flow in small-caliber vessels in various organs. Conventional Doppler employs a unidimensional wall filter to remove background clutter noise artifacts, but in doing so sacrifices signal from slow flow. Through the use of a multidimensional wall filter, MVI removes background clutter while preserving that signal.

Initial studies with MVI focused on differentiating benign masses from tumors, evaluating gonadal torsion, or confirming active rheumatoid arthritis [1]. MVI has recently been applied in adults and children to evaluate the cerebral microvasculature in healthy and ill patients [2,3,4]. Few studies have reported the feasibility of using MVI in preterm and term neonates for characterizing normal cerebral microvascular morphology [5]. Goeral and colleagues evaluated MVI performance in 19 healthy term-born neonates, demonstrating that MVI was superior to color Doppler for depicting anatomical detail and density of the cerebral microvessels, especially those in the extrastriatal (i.e., cortical and medullary) and striatal regions [3]. Similarly, Barletta and colleagues explored the use of MVI in the brain of 15 preterm neonates during the first day of life to detect extrastriatal (cortical or medullary), striatal, or thalamic microvessels [2].

Cerebral MVI could be useful to evaluate various pathologies in neonates and infants. Hwang and colleagues reported preliminary data on several clinical applications of pediatric cerebral MVI for evaluating preterm injury, hypoxic-ischemic injury, stroke and cerebrovascular lesions, brain tumors, infections, hydrocephalus, and patients on extracorporeal membrane oxygenation (ECMO) [5,6]. Previous studies have demonstrated the utility of MVI in evaluating nonvascular anatomical structures. For instance, Kim et al. used MVI to detect the grade of vesicoureteral reflux and direction of urinary flow in children with a history of urinary tract infection compared to the gold-standard voiding cystourethrogram [7]. Hwang and colleagues showed that MVI can detect cerebrospinal fluid (CSF) flow in two infants with post-hemorrhagic hydrocephalus [8]. Specifically, the group used the B-Flow MVI technology (General Electric Healthcare, Inc) to detect CSF flow in the cerebral aqueduct, third, and fourth ventricle. In light of these findings, MVI technology may help assess flow in not only the vasculature of the infant’s brain and other intracranial structures but also the ventricular system [5,8]. In this study, we aim to describe the detection and characterization of intraventricular CSF flow dynamics by MVI in a large retrospective cohort of infants.

## 2. Materials and Methods

### 2.1. Study Design and Participants

This retrospective, cross-sectional, single-center study was approved by our Institutional Review Board. A waiver for informed consent was granted. We searched for electronic records of children who underwent brain ultrasounds performed by a pediatric sonographer with broad experience in MVI (BK) over a 13-month period between September 2020 and September 2021. We used Illuminate InSight™ (Overland Park, KS, USA) software for our initial search. We included infants younger than 1 year of age with available MVI B-Flow cine clips. We excluded low-quality studies (e.g., images that were obscured or not in the sagittal plane). Demographic and clinical data were extracted from patient charts using the EPIC system (Epic Systems Corporation, Verona, WI, USA). The Strengthening the Reporting of Observational Studies in Epidemiology (STROBE) guidelines were used to prepare this manuscript [9].

### 2.2. Exam Protocol

An L2-9 probe and LOGIQ^TM^ E10 (General Electric, Milwaukee, WI, USA) ultrasound scanner were used for image acquisition; grayscale images were acquired in the coronal and sagittal planes through the patent anterior fontanelle; bilateral coronal oblique images were also obtained. B-Flow/MVI was already integrated by the vendor in the US machine. Mid-sagittal B-Flow cine clips lasting approximately 5–10 seconds were obtained.

### 2.3. Image Interpretation and Data Collection

Images were accessed from the IntelliSpace PACS Enterprise 4.5 system (Philips Healthcare, Best, The Netherlands). Two pediatric radiologists (one attending physician [RD] and one in-training fellow [LO]), blinded to reports and each other’s evaluations, individually examined the images (grayscale and MVI B-Flow cine clips) to determine the following: (1) diagnostic impression, including normal brain, hydrocephalus, intraventricular hemorrhage, and other conditions; (2) visualization of CSF flow in the ventricular system, including the third ventricle, the cerebral aqueduct, and the fourth ventricle; and (3) the direction of the CSF flow, which could be craniocaudal, caudocranial, or bidirectional. A third pediatric radiologist (MH), blinded to the responses of the first two, evaluated the discrepancies and determined the final responses. We collected clinical and radiological data using REDCap 10.9.0 (Vanderbilt University, Nashville, TN, USA) [10].

### 2.4. Statistical Analysis

We used IBM SPSS Statistics version 26.0 for statistical analysis. Demographics are presented as mean ± standard deviation (SD). Descriptive data are reported as frequencies and percentages. We used chi-square tests to determine associations between diagnostic impression and visualization of CSF flow in MVI B-Flow. We calculated the inter-rater reliability (IRR) using Cohen’s coefficient Kappa; the level of agreement was considered as none (0–0.2), minimal (0.21–0.39), weak (0.40–0.59), moderate (0.60–0.79), strong (0.80–0.90), and almost perfect (>0.90) [11]. A *p*-value < 0.05 was defined as the level of significance. We used the Holm-Bonferroni method for correction of multiple comparisons.

## 3. Results 

A total of 309 scans were identified after the first search. One-hundred-and-one patients fulfilled the eligibility criteria and were included in the analysis, of which 45 (44.6%) were female. The mean age was 40 ± 53 days, with 60.4% (n = 61) being preterm infants. Of these 61 preterm infants, 45.9% (n = 28) were extremely preterm, 26.2% (n = 16) were very preterm, and 27.9% (n = 17) were moderate-to-late preterm according to the World Health Organization classification. Thirteen patients passed away 82 ± 69 days (mean ± SD) after the brain examination.

A total of 40/101 (39.6%) patients had a normal brain US scan; 9/101 (8.9%) were not interpreted as normal but did not have either hydrocephalus or presence of remarkable intraventricular bleeding and thus were combined into the group “no hydrocephalus or intraventricular hemorrhage” [No H/IVH]. The anatomic abnormalities for this group were: grade I germinal matrix hemorrhage (n = 3), absence/dysgenesis of corpus callosum (n = 2), extra-axial subdural collections (n = 3), mild ventricular prominence (n = 2), and periventricular hemorrhage (n = 1); some patients had more than one of these conditions.

Forty patients (39.6%) had hydrocephalus (26 without IVH), 26 (25.8%) had IVH (12 without hydrocephalus), and 14 (13.9%) had hydrocephalus+IVH. Patients in the group No H/IVH were more likely to have been born at term (*p* = 0.007). Patients with hydrocephalus alone were more likely to be preterm (*p* = 0.004). However, after the Holm–Bonferroni correction, these two apparent associations turned out to be non-significant. There was no significant difference between term and preterm infants in the presence of IVH (*p* = 0.285) or hydrocephalus+IVH (*p* = 0.134) in our cohort. There were no significant differences between the subclassification of preterm patients and the diagnostic impression from US.

**Figure 1:**
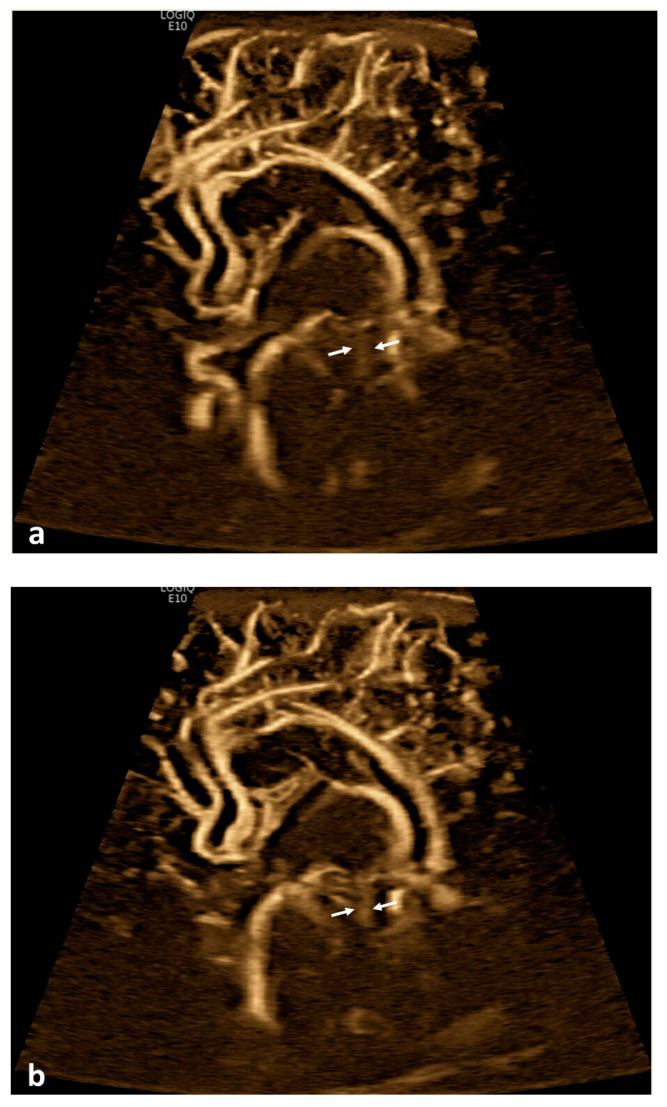

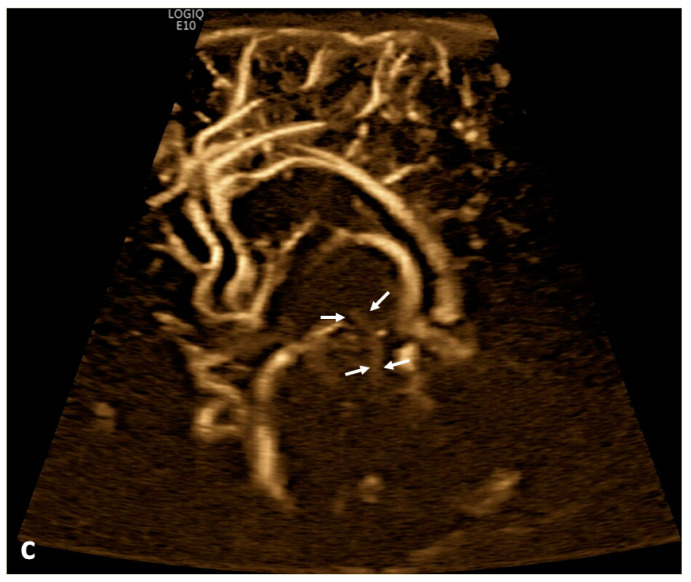
Cerebrospinal fluid (CSF) flow visualized in the ventricular system. A 15-day-old term girl with a history of bilateral grade III intraventricular hemorrhage and hydrocephalus underwent brain ultrasound. This patient also had cardiovascular comorbidities, including dextro-transposition of great arteries, ventricular septal defect, and tricuspid regurgitation. (**a**–**c**) Sagittal B-Flow demonstrates turbulent caudocranial CSF flow in the cerebral aqueduct (arrows). The entire clip is provided as Supplemental Material S1.

**Figure 2:**
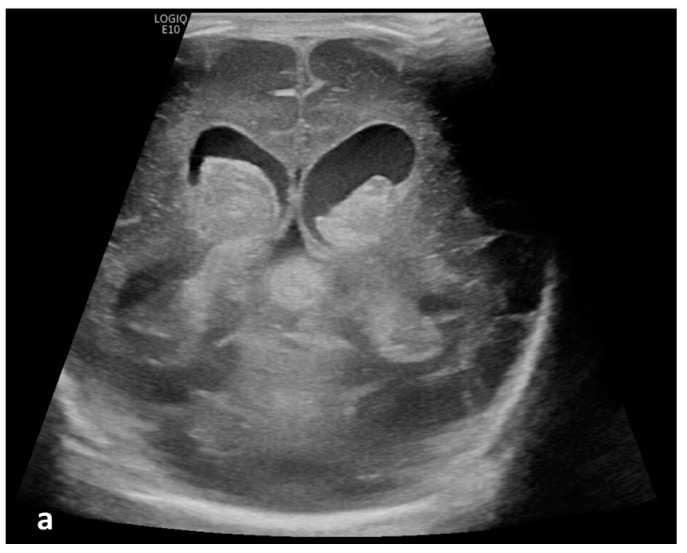

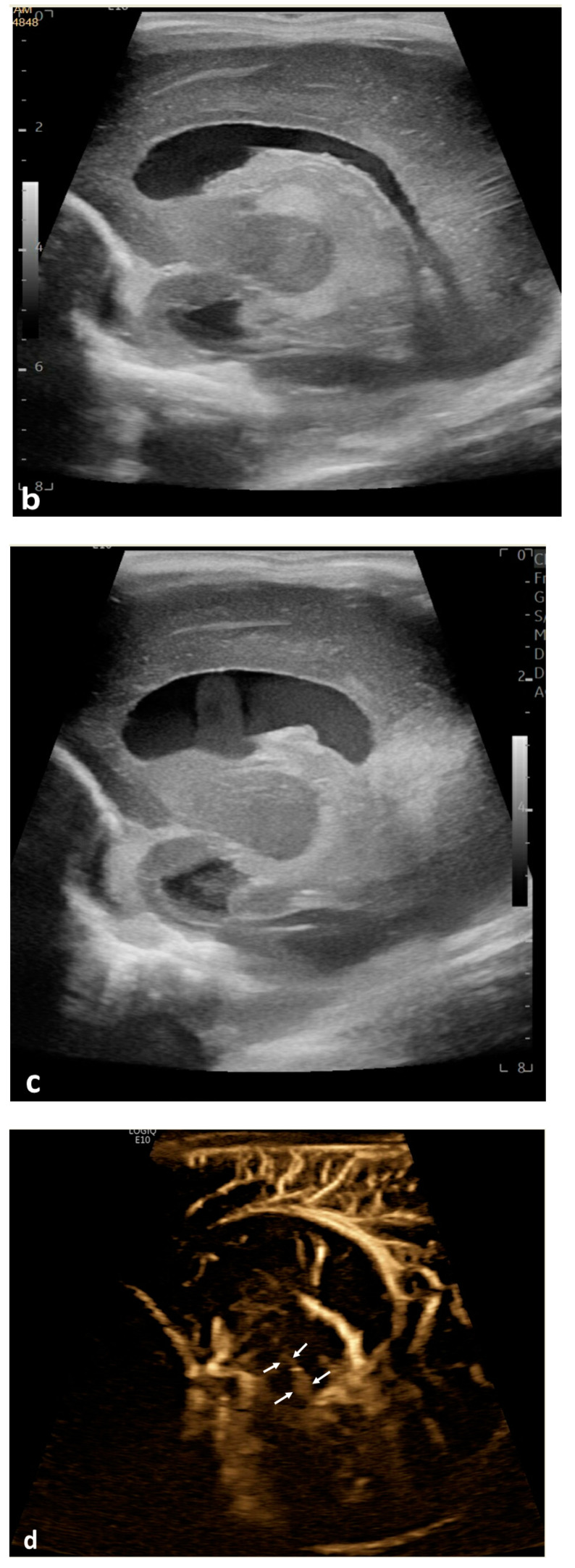

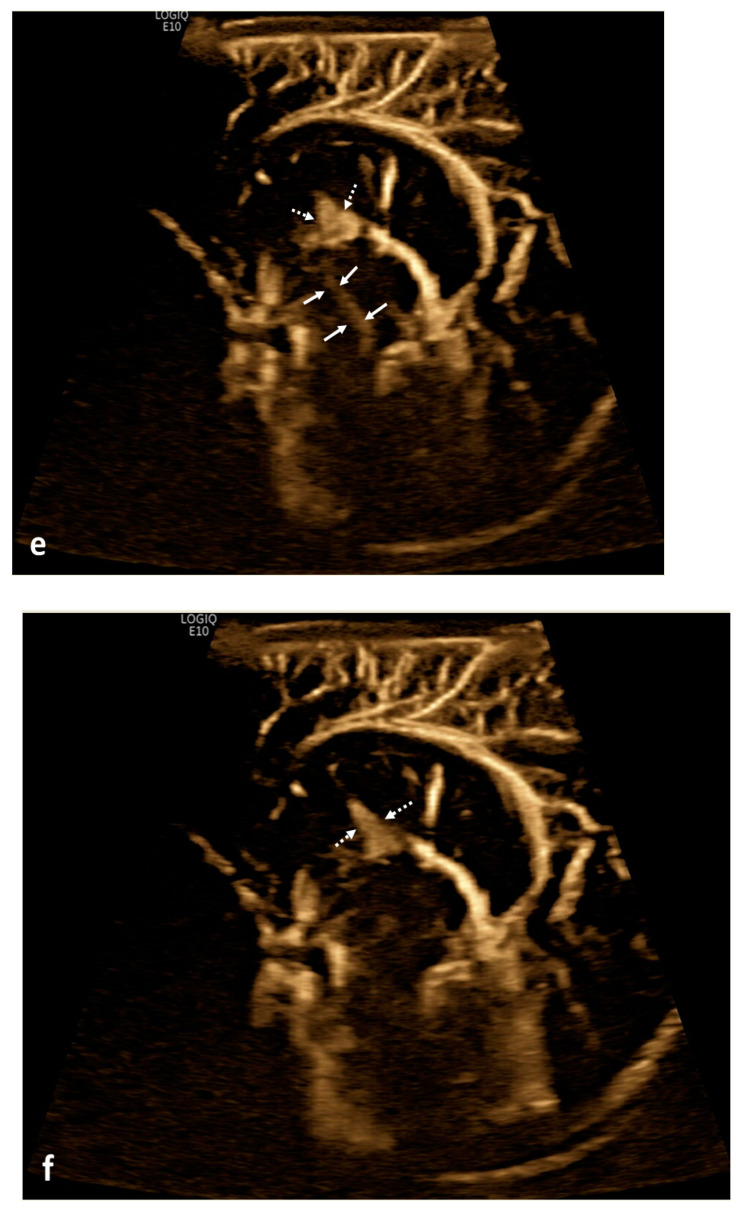
Cerebrospinal fluid (CSF) flow visualized in a patient with post-hemorrhagic hydrocephalus. A 7-day-old boy with a history of severe intraventricular hemorrhage and ventriculomegaly. Coronal and sagittal grayscale ultrasound shows enlargement in the lateral and third ventricles, as well as evolving choroid plexus hemorrhage and fine granular mobile debris in the ventricles (**a**–**c**). The parenchymal evaluation shows grade IV left parieto-occipital periventricular hemorrhage (**c**). Sagittal B-Flow (**d**–**f**) demonstrates turbulent caudocranial CSF flow from the cerebral aqueduct (arrows) into the third ventricle (dotted arrows). The entire clip is provided as Supplemental Material S2.

Twenty patients (19.8%) demonstrated apparent CSF flow detectable using MVI in at least one of the ventricular structures: 11 (10.9%) in the third ventricle, 16 (15.8%) in the cerebral aqueduct, and 17 (16.8%) in the fourth ventricle (Figure 1 and Figure 2). Of these 20 patients, 13 had IVH as visualized in grayscale US; flow direction was detected as craniocaudal in 15% (n = 3) of patients, caudocranial in 70% (n = 14), and bidirectional in 15% (n = 3) (Supplemental S1 and S2; Table 1). The IRR for detection of CSF flow was 0.662, *p* < 0.001. 

Visualization of CSF flow was significantly associated with the presence of IVH alone (OR 9.7 [3.3–29.0], *p* < 0.001) and in the group of hydrocephalus+IVH (OR 12.4 [3.5–440], *p* < 0.001), but not with hydrocephalus alone (*p* = 0.116). IVH alone and the group hydrocephalus+IVH was also significantly associated with presence of flow in each ventricular structure. Comparisons between specific ventricular anatomic subdivisions are presented in Table 2. 

CSF flow was detected in five cases without apparent hydrocephalus or IVH, as detected with grayscale ultrasound. The first patient was an 80-day-old preterm male (ex-27 weeks) who had a ventriculoperitoneal shunt at 53 days of age due to congenital ventriculomegaly; however, ventricular size was interpreted as normal at the time of the scan; CSF flow was seen as caudocranial in the cerebral aqueduct. Other associated conditions included a history of placental abruption and anemia. A brain magnetic resonance imaging (MRI) scan 4 months later demonstrated an indwelling ventriculostomy catheter and decompressed ventricles. Susceptibility weighted imaging (SWI) revealed findings in keeping with a prior IVH along the lateral aspect of the left lateral ventricle and within the left choroid plexus; therefore, even though this case was indeed IVH, ultrasound was not sensitive enough to detect blood products. The second patient was a 3-day-old term female on ECMO due to meconium aspiration syndrome and persistent pulmonary hypertension. Brain parenchyma demonstrated diffuse heterogeneous hyperechogenicity of the white matter and an echogenic focus in the left thalamus; CSF flow was interpreted as bidirectional in the fourth ventricle. A brain MRI 7 days later demonstrated several punctate foci of signal abnormality in the right periatrial white matter and left frontal lobe, representing white matter injury, without hydrocephalus or IVH.

The third patient was an 11-day-old preterm male (ex-34 weeks) with a history of intrauterine growth restriction and gastroschisis; brain US was indicated to rule out intracranial hemorrhage due to prematurity but was interpreted as normal; CSF flow was seen as craniocaudal in the cerebral aqueduct and the fourth ventricle. No additional follow-up neuroimaging studies were obtained. The fourth patient was a 1-day-old preterm twin female (ex-35 weeks) with a history of perinatal depression who was placed on therapeutic hypothermia for suspicion of hypoxic-ischemic encephalopathy; CSF flow was interpreted as caudocranial in the third ventricle. MRI performed 5 days later was normal. The fifth patient was a 27-day-old preterm male (ex-24 weeks) with a history of genitourinary malformations, chronic lung disease, feeding intolerance and glucose-6-phosphate dehydrogenase deficiency; CSF flow was caudocranial in the fourth ventricle. Follow-up brain US scans were also unremarkable after 1, 2, and 7 months.

## 4. Discussion 

**Figure 3:**
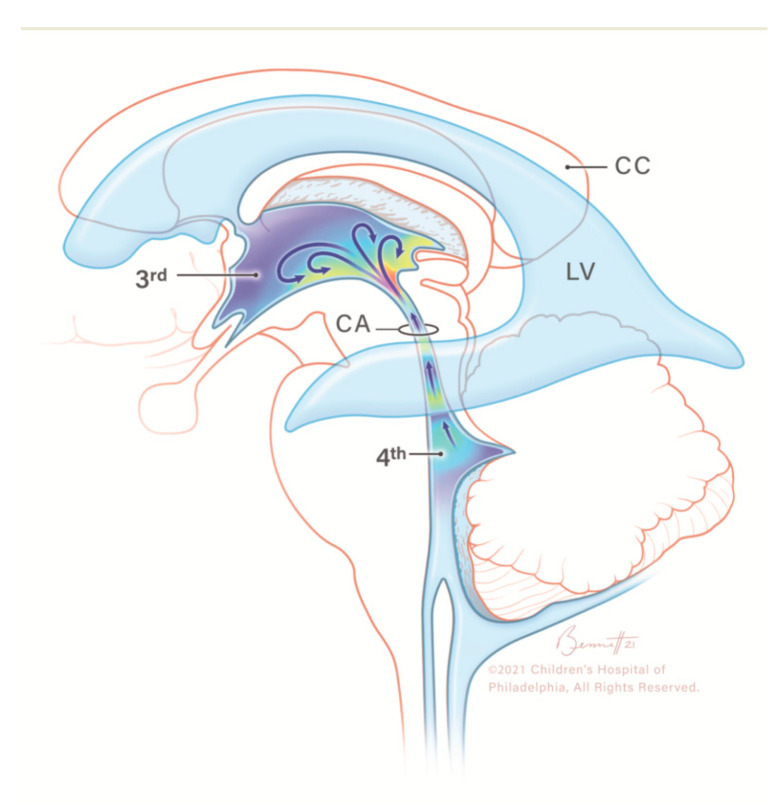
Illustration of caudocranial cerebrospinal fluid (CSF) flow direction, circulating from the fourth ventricle, passing through the cerebral aqueduct into the third ventricle (arrows). CC, corpus callosum; CSF, cerebrospinal fluid; LV, lateral ventricle; 3rd, third ventricle; CA, cerebral aqueduct; 4th, fourth ventricle. Modified from Hwang et al. with permission.

The evolving research on US systems has developed advanced Doppler technologies, such as MVI, that help depict flow in microvessels as well as in extravascular structures. Hwang and colleagues reported for the first time that CSF flow could be visualized in two neonates with a history of post-hemorrhagic hydrocephalus [8] (Figure 3). We found that using MVI, CSF flow could be identified in neonates and infants with different conditions, including hydrocephalus, IVH, or in some brains without apparent hydrocephalus or bleeding. Furthermore, there was a significant association between CSF flow visualization and an underlying diagnosis of IVH, either in the presence or absence of hydrocephalus.

CSF is produced mainly in the choroid plexus of the lateral ventricles and plays a role in protecting encephalic structures from mechanical injury, transporting nutrients, and removing toxins/waste products from the neural tissue [12,13]. CSF flow depends on cardiac pulsations and respiratory cycles, and its flow and composition can be altered under pathological conditions [12,14]. CSF is typically thought to travel craniocaudally, from the paired lateral ventricles to the third ventricle through the foramen of Monro, then to the fourth ventricle through the cerebral aqueduct of Sylvius before reaching the subarachnoid space through the foramina of Magendie and Luschka [12]. There are several proposed mechanisms that can help elucidate CSF flow within the ventricular system, including the coordinated pulsatile movement generated by the choroid plexus and the ependymal cilia, in combination with pressure waves produced by arterial blood flow that facilitate CSF’s passage through the ventricular system [15]. Few studies have elucidated that CSF flow dynamics in the pediatric population might be different from those seen in adults; for instance, children might present minimal variations in the cross-sectional areas of the cerebral aqueduct when compared to adults, which could explain the independency of CSF parameters when compared to biometric values in children [16,17,18,19,20].

CSF dynamics can be studied using different MRI methods, including phase-contrast and time spatial inversion pulse [21,22]. Nevertheless, MRI is still a relatively expensive modality with limited availability in most settings around the world, which may be disadvantageous in emergent situations; furthermore, MRI poses a need of sedation in young children that is not innocuous and may involve injection of exogenous material into the CSF space [23,24]. Nowosławska and colleagues used phase-contrast MRI to measure the pulsatile CSF flow parameters in two different compartments (Sylvian aqueduct and prepontine cistern) of children with ventriculomegaly to predict the risk of acute hydrocephalus. Children with hyper-oscillating flow through these structures tended to be more symptomatic, presenting with increased head circumference growth rate, paresis, signs of chronic increased intracranial pressure, mental retardation, headaches, and/or epileptic seizures [23]. In their prospective study, Öztürk and colleagues established normal values of CSF flow parameters (velocity, volume, and aqueduct area) using phase-contrast MRI in infants, children, and adolescents. They showed significant differences in specific CSF flow parameters across certain groups of ages and between males and females. Specifically for infants, there was a statistically significant difference between infants and children in regards to cranial direction volume (0.01 mL vs. 0.02 mL), but there were no differences in respect to peak velocity, average velocity, caudal direction volume, net volume, or aqueductal area between infants and other age categories [25]. Of note, Plata and colleagues used cine phase-contrast MRI to obtain normal CSF measurements in the aqueduct of healthy children, including net flow, stroke volume, and volume velocity, among others; these values were independent of age, sex, or body surface area [17]. Table 3 summarizes the various current and emergent imaging protocols used for the evaluation of CSF dynamics in humans [24,26,27,28,29,30,31].

Previous studies have used ultrasound to evaluate CSF flow in children with intraventricular hemorrhage. For example, Tatsuno and colleagues studied the CSF flow dynamics in 6 infants using color Doppler flow imaging through the anterior fontanelle and were able to visualize the flow in the cerebral aqueduct, the third ventricle, and the foramina of Monro, as well as measure velocities. They found that flow was caudocranial from mid-inspiration to mid-expiration whereas it was craniocaudal from mid-expiration to mid-inspiration [14]. However, this is the first study using advanced Doppler technology that demonstrates CSF flow in infants with or without history of intraventricular bleeding in a large cohort. Moreover, we sought to validate the utility of a flow-based technology in detecting CSF dynamics in a cohort oh healthy and diseased patients. Although we did not perform dynamic interventions, this concept could be expanded in future research on MVI applications. 

In our study, most of the patients in which CSF flow was demonstrated had a caudocranial flow direction, which is consistent with other studies that conclude that CSF flow is not necessarily craniocaudal but can present as bidirectional [12,32]. These physiologic differences at early ages might have an impact in CSF flow dynamics that can ultimately manifest as caudocranial or bidirectional flow, as seen in our cohort, even in the absence of blood products [12,20,33]. In patients with post-hemorrhagic hydrocephalus, moreover, blood products can serve as a mechanical obstruction for craniocaudal flow. The CSF flow directionality may have both prognostic and therapeutic implications, as the restoration of normal CSF flow is likely important for brain health in infants. 

Post-hemorrhagic hydrocephalus occurs mostly in preterm infants after lysis of red blood cells that triggers a pro-inflammatory response and eventual periventricular white matter injury; this generates the release of degradation bioproducts such as ferritin, hemoglobin, and bilirubin particles [34,35]. It is unclear whether the particles that reflect signal on ultrasound are red blood cells or their degradation bioproducts, especially considering previous studies on pediatric vesicoureteral reflux, where flow direction has also been detected in the setting of urinary tract infection [7]. This highlights the importance of the presence of debris/particles that alter fluids’ composition, viscosity, and turbulence for detecting any flow [36]; in light of this, other neurological conditions that modify CSF physicochemical properties (e.g., meningitis, ventriculitis) may also allow visual recognition of CSF ventricular flow although this warrants further studies.

Interestingly, four out of five patients without apparent hydrocephalus or IVH in ultrasound, in which CSF flow was visualized, were preterm infants. The fifth patient was born at term but had a complex medical history, including being on ECMO. After reviewing these patients’ clinical charts, only two (one term and one preterm) had a history of bleeding or white matter injury. We hypothesize that the complex and serious medical histories of these patients (i.e., prematurity and intrauterine growth restriction, major cardiopulmonary disturbances requiring ECMO, post-surgical status, and hypoxic-ischemic encephalopathy) may have predisposed to a change in CSF normal characteristics and composition by altering the intracranial intra- and extravascular autoregulation mechanisms [37], ultimately allowing the detection of CSF flow in the absence of IVH. Other explanations could be related with altered vascular pulsatility that affects CSF pulsation, CSF clearance, and CSF compliance. Finally, there may have been trace blood products not imaged or detected.

CSF MVI could be potentially applied as an adjunctive tool for diagnosing or following-up infants with suspicion of post-hemorrhagic hydrocephalus. Since this technology is commonly integrated in the ultrasound machine, its use would not add a financial or logistic burden during image acquisition. MVI, in combination with grayscale and Doppler ultrasound, could enhance the clinical significance of serial brain ultrasounds; it could even be tested to assess real-time changes in CSF flow after shunt placement. The visualization of CSF using MVI could have important management and/or prognostic value if correlated with outcomes in the future. 

This study has some limitations that need to be addressed. First, its retrospective nature poses an inherent risk of selection bias, considering that all scans were acquired by a single sonographer, the lead expert in MVI at our institution. Second, the sensitivity of some studies during imaging acquisition may have been altered due to motion, producing flash artifacts. Third, the diagnostic accuracy of MVI technology varies depending on the vendor and probe settings, limiting reproducibility if implemented with different vendors [8]. Lastly, in the present study, we did not consider whether patients with hydrocephalus and/or IVH had undergone shunt placement or the degree of severity of these conditions, which would be interesting to explore in further studies.

In conclusion, this study demonstrates that MVI allows the evaluation of qualitative CSF flow dynamics in infants, with a substantial inter-rater agreement, especially in the setting of suspected IVH. This nonvascular application of MVI US warrants further investigation to better determine this technique’s sensitivity for CSF detection in various conditions, considering CSF composition, turbulence, and velocity. The clinical implications of non-invasive, real-time visualization of CSF dynamics are significant, as alterations in CSF dynamics have been associated with pathogenesis and outcomes of various neurologic diseases affecting infants, including post-hemorrhagic hydrocephalus.

## Figures and Tables

**Table 1:** Identification of CSF flow in infants using MVI technology.

	CSF Flow (%)	Third Ventricle (%)	Cerebral Aqueduct (%)	Fourth Ventricle (%)	CSF Flow direction		
					Craniocaudal (%)	Caudocranial (%)	Bidirectional (%)
Hydrocephalus * (n = 40)	11 (27.5%)	7 (17.5%)	10 (25%)	11 (27.5%)	2 (5%)	8 (20%)	1 (2.5%)
IVH ^+^ (n = 26)	13 (50%)	10 (38.5%)	12 (46.2%)	12 (46.2%)	1 (3.8%)	11 (42.3%)	1 (3.8%)
Hydrocephalus+IVH (n = 14)	9 (64.3%)	7 (50%)	8 (57.1%)	9 (64.3%)	1 (7.1%)	8 (57.1%)	0 (0%)
Non hydrocephalus, non IVH (n = 49)	5 (10.2%)	1 (2%)	2 (4.1%)	3 (6.1%)	1 (2%)	3 (6.1%)	1 (2%)

CSF: cerebrospinal fluid; IVH: intraventricular hemorrhage; MVI: microvascular imaging. A total of 20 patients showed CSF flow in MVI and belonged to the following categories: hydrocephalus+IVH (n = 9), No H/IVH (n = 5), IVH without hydrocephalus (n = 4), and hydrocephalus without IVH (n = 2). * Included patients with hydrocephalus, with or without IVH. ^+^ Included patients with IVH, with or without hydrocephalus.

**Table 2:** Detection of CSF flow between groups.

	Evidence of CSF Flow [OR (CI)]							
	Any Structure	*p*-Value	Third Ventricle	*p*-Value	Cerebral Aqueduct	*p*-Value	Fourth Ventricle	*p*-Value
Hydrocephalus	2.2 (0.8–5.9)	0.116	3.0 (0.8–11.1)	0.084	3.1 (1.0–9.2)	0.041	3.5 (1.2–10.4)	0.02
IVH	9.7 (3.3–29.0)	0.000007 *	46.3 (5.5–387.4)	0.0000002 *	15.2 (4.3–54.1)	0.0000009 *	12 (3.6–39.5)	0.000004 *
Hydrocephalus+IVH	12.4 (3.5–44.0)	0.000007 *	20.8 (4.9–88.5)	0.0000004 *	13.2 (3.6–47.6)	0.000005 *	17.8 (4.8–66.1)	0.0000003 *
No H/IVH	0.28 (0.09–0.84)	0.019	0.09 (0.01–0.71)	0.006 *	0.12 (0.03–0.54)	0.002 *	0.18 (0.05–0.66)	0.005 *

CSF: cerebrospinal fluid; IVH: intraventricular hemorrhage; OR: odds ratio: CI: confidence interval; No H/IVH: no hydrocephalus, no IVH. * Remained significant after Holm–Bonferroni correction for multiple comparisons.

**Table 3:** Current and emergent imaging protocols used for the evaluation of cerebrospinal fluid flow.

Modality	Protocol	Uses and Advantages	Shortcomings
CT	Cisternography/Myelography [31]	- Identification of CSF leakage, CSF flow tracking, and spinal canal and cord anatomy.	- Uses ionizing radiation. - May need sedation in young children. - Often uses intrathecal injection of contrast with potential neurotoxicity. - Limited evaluation of CSF flow dynamics and velocity.
MRI	Cisternography/Myelography [26,27]	- Identification of CSF leakage, CSF flow tracking, and spinal canal and cord anatomy.	- May need sedation in young children. - Often uses intrathecal injection of contrast with potential neurotoxicity. - Limited evaluation of CSF flow dynamics and velocity.
MRI	Phase-contrast MRI^+^ [27,28,29]	- Qualitative and quantitative (e.g., stroke volume, net flow, peak velocity, mean velocity) evaluation of CSF flow and velocity throughout the cardiac cycle.	- May need sedation in young children. - Works in plane perpendicular to flow. - Errors in velocity encoding value can cause aliasing or reduced flow sensitivity. - Susceptible to artifact. - One measurement is average of multiple cardiac cycles.
MRI	Time-SLIP [28,30]	- Visualization of CSF bulk and turbulent flow, independent of the cardiac cycle. - Superior anatomic detail to phase-contrast.	- May need sedation in young children. - Data acquisition limited to short periods (seconds).
US	MVI [8]	- Real-time visualization of CSF flow direction.	- Operator dependent. - Decreased spatial resolution compared to MRI. - Visualization of CSF in select conditions (i.e. intraventricular hemorrhage and hydrocephalus).
PET	Dynamic PET [27]	- Visualization of CSF flow and clearance.	- Use of radiotracer. - May need sedation in young children. - Suboptimal anatomic imaging unless combined with CT or MRI.
NM	Cisternography [24]	- Visualization of CSF leaks and evaluation of hydrocephalus.	- Use of radiotracer. - May need sedation in young children. - Suboptimal anatomic imaging.

MRI: magnetic resonance imaging; CSF: cerebrospinal fluid; Time-SLIP: time-spatial labeling inversion pulse; MVI: microvascular imaging ultrasound; PET: positron emission tomography; NM: nuclear medicine. + Useful sequence to follow-up after third ventriculostomy.

## Data Availability

Data are available from the corresponding author upon reasonable request.

## Supplementary Materials

The following supporting information can be downloaded at: https://www.mdpi.com/article/10.3390/children10020245/s1, Supplement S1. Clip of visualized caudocranial cerebrospinal fluid flow in the cerebral aqueduct in a 15-day-old term girl with a history of bilateral grade III intraventricular hemorrhage. Supplement S2. Clip of visualized caudocranial cerebrospinal fluid flow in the cerebral aqueduct entering the third ventricle in a 7-day-old term boy with a history of severe intraventricular hemorrhage and ventriculomegaly.
